# Linezolid Resistance in *Enterococcus faecalis* Associated With Urinary Tract Infections of Patients in a Tertiary Hospitals in China: Resistance Mechanisms, Virulence, and Risk Factors

**DOI:** 10.3389/fpubh.2021.570650

**Published:** 2021-02-05

**Authors:** Xiaoyu Ma, Fan Zhang, Bing Bai, Zhiwei Lin, Guangjian Xu, Zhong Chen, Xiang Sun, Jinxin Zheng, Qiwen Deng, Zhijian Yu

**Affiliations:** ^1^Department of Infectious Diseases and Quality Control Center of Hospital Infection Management of Shenzhen, Shenzhen Nanshan People's Hospital and the 6th Affiliated Hospital of Shenzhen University Health Science Center, Shenzhen, China; ^2^Shenzhen Key Laboratory for Endogenous Infections, Guang Dong Medical University, Shenzhen, China; ^3^Department of Tuberculosis, Shenzhen Nanshan Center for Chronic Disease Control, Shenzhen, China

**Keywords:** linezolid resistance, urinary tract infection, virulence factor, resistance genes, *Enterococcus faecalis*

## Abstract

**Background:** *Enterococcus faecalis* has been commonly considered as one of the major pathogens of the urinary tract infection (UTI) in human host worldwide, whereas the molecular characteristics of *E. faecalis* clinical isolates from the patients with UTI in China remains seldomly reported. This study aimed to investigate the resistance mechanism, molecular characteristics and risk factors of *E. faecalis* clinical isolates from patients with UTI in China.

**Methods:** A total of 115 non-duplicated *E. faecalis* clinical isolates from patients with UTI were retrospectively collected in a tertiary hospital in China and their clinical data was further analyzed. The linezolid and tedizolid susceptibility were determined by agar dilution. The resistance genes, including *erm*(A)*, erm*(B)*, erm*(C)*, tet*(M)*, optrA, cfr, cfr*(B), *poxtA*, and MLST-based housekeeping genes were investigated by PCR.

**Results:** In 115 non-duplicated *E. faecalis* clinical isolates from the patients with UTI in this hospital setting, the frequency of linezolid or tedizolid-resistant/intermediate isolates were 22.61 and 13.04%, respectively, and the frequency of linezolid-resistant/intermediate *E. faecalis* clinical isolates carrying with *erm*(A) were 86%. Among the five linezolid-resistant *E. faecalis* strains found in this study, three *optrA-*positive isolates and the other two linezolid-resistant strains were G2576U genetic mutations in the V domain of the 23S rRNA genes. The ST clonality analysis indicated that 31.42% (11/35) of ST16 *E. faecalis* UTI isolates were not susceptible to linezolid. Moreover, the univariable analysis indicated that the high risk factors of linezolid-resistant/intermediate *E. faecalis* infections involved the indwelling catheter, trachea cannula catheter and the carriage of *erm*(A) or *optrA*. Furthermore, the indwelling catheter and trachea cannula catheter were demonstrated as the independent predictors of linezolid-resistant/intermediate *E. faecalis* strains in patients with UTI by multivariable analysis.

**Conclusion:** Linezolid-resistant/intermediate *E. faecalis* associated with urinary tract infections of patients in this hospital setting from China might be explained by the high carriage frequency of *optrA* genes and moreover, indwelling catheter and trachea cannula should be considered as the independent predictors of linezolid-resistant/intermediate *E. faecalis* infections. The transmission mechanism of linezolid-resistant/intermediate *E. faecalis* in this hospital setting should be further studied.

## Introduction

*Enterococcus faecalis* has been widely considered as the commensal inhabitants of the intestinal tract of both humans and animals (1). *E. faecalis* is the most prevalent species of *Enterococcus* genus that is isolated from the clinical specimens among human hosts with a series of infectious diseases, such as sepsis, abdominal infections, endocarditis, cholecystitis, peritonitis, and neonatal meningitis (2). Moreover, *E. faecalis* has been regarded as one of the major pathogens from patients with the urinary tract infection (UTI) in clinics worldwide (3, 4). Because of the inherent resistance of *E. faecalis* to several antibiotic agents and their natural competence for acquired resistance, the treatment difficulty of *E. faecalis* infections has gradually increased in recent years (5). Linezolid, the first synthetic antimicrobial agent of oxazolidinone class, inhibits the initial ribosome assembly and protein synthesis of multiple gram-positive bacteria species by targeting the 50S ribosome subunits and impacting its binding affinity with formylmethionyl-tRNA (6). Due to its broad antimicrobial spectrum, linezolid has been widely used as one of the most important options for the treatment of infectious diseases caused by multi-drug resistant gram-positive pathogens, especially including methicillin-resistant *Staphylococcus aureus* (MRSA), vancomycin-resistant enterococci (VRE), penicillin-resistant streptococci and mycobacteria (7). In recent years, with the widespread application of linezolid in clinics, the gradual increasing reports of linezolid resistant gram-positive pathogens highlights the enhanced risk of linezolid resistance transmission (8, 9). Our previous data indicated the possible presence of high frequency of linezolid resistance in *E. faecalis* clinical isolates. However, the frequency and clinical significance of linezolid-resistant/intermediate *E. faecalis* in patients with UTI remains elusive (10).

In this study, the *E. faecalis* clinical isolates from the patients with UTI were collected from a tertiary hospital in China. Subsequently, the clinical data of the patients with UTI was further analyzed. The antimicrobial susceptibility of linezolid and tedizolid was determined by agar dilution. The resistance genes, including *erm*(A), *erm*(B), *erm*(C), *tet*(M), the domain V region of the 23S rRNA gene, *cfr, cfr*(B), *poxtA*, as well as *optrA* and several commonly detected virulence factors were investigated by PCR. The ST genotype was determined by detecting MLST-based housekeeping genes and their relationship with linezolid-resistant/intermediate *E. faecalis* infections was further analyzed.

## Materials and Methods

### Bacterial Isolates and Patients Clinical Data

A total of 115 non-duplicate clinical *E. faecalis* UTI isolates were collected from January 1, 2010 to September 30, 2015 in Nanshan People's Hospital (A teaching hospital) of Shenzhen, China(It is a grade A class three general hospital located in Nanshan District, Shenzhen, with more than 1,300 open beds). *E. faecalis* clinical strains were isolated from the urine samples and identified by the VITEK 2 system (BioMérieux, Marcyl'Etoile, France). Species-appropriate quality control strains were used to ensure laboratory standards, as directed by the Clinical and Laboratory Standards Institute (CLSI 2020) (11). *E. faecalis* ATCC29212 and OG1RF (ATCC47077), obtained from the American Type Culture Collection (ATCC), were used as quality control strains. Patient clinical data including age, gender, admission to intensive care unit (ICU), venous catheter, indwelling catheter, D-J tube catheter, trachea cannula catheter and antibiotics therapy, were collected from hospital information system. *E. faecalis* clinical isolates of inpatients in Nanshan People's Hospital were analyzed retrospectively and approved by the institutional ethical committee of Shenzhen Nanshan people's hospital. This trial followed the ethical principles of the Declaration of the Chinese Ethical Guidelines. All procedures involving human participants were performed in accordance with the ethical standards of Shenzhen University and the 1964 Helsinki declaration and its later amendments or comparable ethical standards.

### Antibiotic Susceptibility Testing

The antimicrobial susceptibility of several commonly-used antibiotics, such as tetracycline, erythromycin, vancomycin, minocycline, tigecycline, vancomycin, tedizolid, linezolid and doxycycline in *E. faecalis* were automatically tested through VITEK 2 Compact system (BioMérieux, France). The susceptibility breakpoints of these antibiotics in *E. faecalis* were recommended by CLSI 2020 (11). The MIC values of linezolid, tedizolid and tigecycline were further determined by agar dilution according to related reports (10, 12, 13). The linezolid susceptible breakpoint recommended in *E. faecalis* by CLSI was adopted: ≤ 2 μg/mL for susceptibility, 4 μg/mL for intermediate status, and ≥8 μg/mL for resistance. The susceptible breakpoint of tedizolid to *E. faecalis* was defined as MIC ≤ 0.5 μg/mL (11). The MIC breakpoints for tigecycline recommended by the European Committee on Antimicrobial Susceptibility Testing (EUCAST), the strains with MIC > 0.25 μg/mL was classified as resistant to tigecycline (14).

### DNA Extraction and Polymerase Chain Reaction for Detection of Resistance Genes and Virulence Factors

The genomic DNA of the bacteria was extracted by DNeasy Blood & Tissue Kit DNA extraction kit (MGI Tech Co, Ltd, Shenzhen, China) according to the performance procedure of gram-positive bacteria, and the extracted DNA was stored at −20°C. The primers in this study listed in Supplementary Table 1 were synthesized by BGI company (13, 15, 16). PCR was carried out for the detection of the following resistance genes: *erm*(A)*, erm*(B)*, erm*(C)*, tet*(M), the domain V region of the 23S rRNA gene, *cfr, cfr*(B), *poxtA* as well as the ABC transporter *optrA* (13, 15, 16). Several commonly found virulence factors in the *E. faecalis*, including *asal, esp, gelE, cyl, hyl, efaA*, and *ace*, were amplified by PCR based on published documents (17, 18).

### Multilocus Sequence Typing

On the basis of established Multilocus sequence typing (MLST) schemes (http://www.mlst.net), seven housekeeping genes of *E. faecalis* (*gdh, gyd, pstS, gki, aroE, xpt*, and *yiqL*) were amplified and sequenced as described previously and the primers of the housekeeping genes were listed in Supplementary Table 3 (13). Sequence types (STs) were determined by comparison with published locus types in the *E. faecalis* MLST.net database (http://efaecalis.mlst.net/). A. Allelic profile or STs were assigned seven integers, corresponding to the allele numbers at the seven loci. STs were assigned to isolates in such a way that the same ST names were kept as far as possible for the same analyzed strains.

### Statistical Analysis

The prevalence of antibiotic susceptibility among the isolates is presented as the number (percentage). This prevalence was compared between groups using the chi-square test or Fisher's exact test. Univariable and multivariable conditional logistic regression were performed to determine patient characteristics associated with the development of infection. The tests were performed using SPSS software (version 19.0, Chicago, IL, USA). *P* < 0.05 were regarded as statistically significant.

## Results

### Antimicrobial Susceptibility of *E. faecalis* UTI Isolates

The 115 non-duplicated *E. faecalis* clinical isolates were obtained from urine samples in the patients with UTI and the distribution characteristics of *E. facecalis* clinical isolates from the hospital wards was shown in Supplementary Figure 1. Our data indicated that linezolid-resistant/intermediate *E. faecalis* was found in 23% (26/115) of the patients with *E. faecalis* UTI. Only five linezolid-resistant *E. faecalis* were detected with linezolid MIC ≥ 8 μg/mL. The relationship of antibiotic susceptibility between linezolid and several commonly used antibiotics was analyzed in Table 1, indicating the frequency of *E. faecalis* UTI isolates with resistance toward erythromycin, doxycycline, tetracycline and minocycline were 99.13% (114/115), 93.91% (108/115), 93.04% (107/115), and 92.17% (106/115), respectively. Worthy of our concern, although the high frequency of the linezolid-resistant/intermediate *E. faecalis* strains were found, these strains exhibited the susceptibility phenotype toward ampicillin, tigecycline and vancomycin (Supplementary Table 4). Moreover, 13.04%(15/115) of *E. faecalis* clinical isolates in this study were non-susceptible to tedizolid.

**Table 1:** The relationship of linezolid susceptibility and antimicrobial susceptibility of commonly used antibiotics in *E. faecalis* causing UTI.

**Drug**	**Total (N) and Rate (%) Δ**	**MIC breakpoint (μg/mL)**	**N**	**Linezolid MIC level, N (μg/mL)**		
				**≤2**	**4**	**≥8**
Total	115	-	115	89	21	5
Tetracycline	107, 93.04%	≤ 4	8	8	0	0
		8	3	3	0	0
		≥16	104	78	21	5
Doxycycline	108, 93.91%	≤ 4	7	7	0	0
		8	7	7	0	0
		≥16	101	75	21	5
Minocycline	106, 92.17%	≤ 4	9	9	0	0
		8	12	11	1	0
		≥16	94	69	20	5
Erythromycin	114, 99.13%	≤ 0.5	1	1	0	0
		1–4	19	15	4	0
		≥8	95	73	17	5
Tedizolid	15, 13.04%	≤ 0.5	100	84	16	0
		>0.5	15	5	5	5

Δ*Includes the total amount of linezolid-resistant strains and linezolid-intermediate strains*.

### Relationship of Linezolid Resistance Genes and Virulence Factors in *E faecalis* From UTI

**Figure 1:**
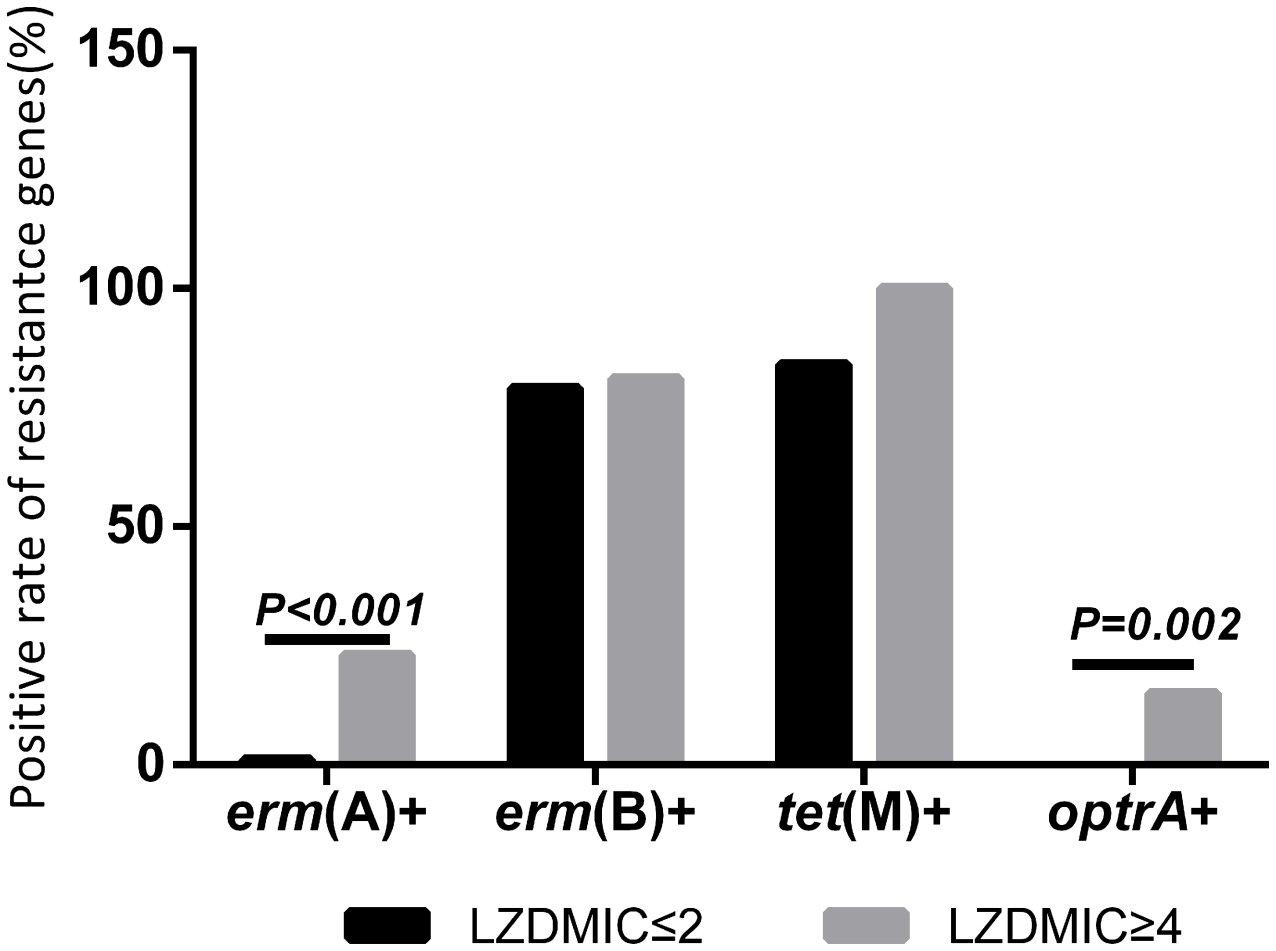
Distribution of the resistance genes [*erm*(A), *erm*(B), *tet*(M), and *optrA*] in *E. faecalis*.

The detection of several resistance genes and virulence factors in this study was shown in Tables 2, 3, Figure 1, and Supplementary Figure 2, indicating that the carriage frequency of the virulence genes, including *esp, hyl, asal, cyl, ace, gelE*, and *efaA*, were 68.70% (79/115), 20.87% (24/115), 83.48% (96/115), 75.65% (87/115), 100% (115 /115), 63.48% (73/115), and 98.26%(113/115), respectively. Moreover, 8.70% of *E. feacalis* UTI isolates were shown with the carriage of all seven virulence factors (*esp*/*efaA*/*asa1*/*ace*/*cyl*/*gelE/hyl*).

**Table 2:** The distribution of the antimicrobial resistance genes and virulence genes in linezolid-Intermediate/Resistant *E. faecalis*.

**No. (rate%)**		**Susceptible isolates MIC distribution (μg/mL)**			**Intermediate/Resistant isolates MIC distribution (μg/mL)**		
		**≤0.5**	**1**	**2**	**4**	**8**	**16**
Total	115	11	30	48	21	4	1
*esp*	79 (68.70%)	7	20	34	15	2	1
*asal*	96 (83.48%)	10	28	38	17	2	1
*hyl*	24 (20.87%)	2	8	9	1	4	0
*cyl*	87 (75.65%)	9	25	33	17	2	1
*gelE*	73 (63.48%)	7	21	33	10	2	0
*efaA*	113(98.26%)	11	30	47	20	4	1
*ace*	115 (100%)	11	30	48	21	4	1
*erm*(A)	7 (6.09%)	1	0	0	1	4	1
*erm*(B)	92 (80%)	10	27	34	16	4	1
*erm*(C)	0	0	0	0	0	0	0
*erm*(A)*+erm*(B)	7 (6.09%)	1	0	0	1	4	1
*erm*(A)*+erm*(C)	0	0	0	0	0	0	0
*erm*(B)*+erm*(C)	0	0	0	0	0	0	0
*tet*(M)	101 (87.83%)	9	27	39	21	4	1
*optrA*	4 (3.48%)	0	0	0	1	2	1
*cfr*	0	0	0	0	0	0	0
*cfr*(B)	0	0	0	0	0	0	0
*poxtA*	0	0	0	0	0	0	0

+*Means exist at the same strain*.

**Table 3:** Characteristics of linezolid in the *optrA*-positive *E. faecalis* strains.

**Strains NO**	***optrA***	**MLST**	**Linezolid MIC (μg/mL)**	***ace***	***efaA***	***asa1***	***cyl***	***esp***	***gelE***	***hyl***	***tet*(M)**	***erm*(A)**	***erm*(B)**	***erm*(C)**	***cfr***	***cfr*(B)**	***poxtA***
EF16C3	+	ST16	16	+	+	+	+	+	–	–	+	+	+	–	–	–	–
EF16C112	+	NT	4	+	+	+	+	+	+	–	+	+	+	–	–	–	–
EF16C299	+	NT	8	+	+	+	+	+	+	+	+	+	+	–	–	–	–
EF16C360	+	ST541	8	+	+	–	–	–	–	+	+	+	+	–	–	–	–

Eighty percent of *E*. *faecalis* UTI isolates in this study were found positively with *erm*(B), but only 7 strains (6.09%) were carried positively with *erm*(A), and no *erm*(C) gene was found. Moreover, our data indicated 68.53% (61/89) of *E. faecalis* UTI isolates with *gelE* was susceptible to linezolid, which was significantly high in comparison to that in the *gelE*-negative isolates. Furthermore, the frequency of the linezolid-resistant/intermediate *E. faecalis* with *erm*(A) was 86%, indicating the high frequency of this resistance genes in linezolid-resistant/intermediate *E. faecalis* UTI isolates (Figure 1 and Supplementary Figure 2).

### Linezolid Resistance Mechanism and Relationship of Linezolid Susceptibility and the ST Genotype

The plasmid-borne resistance genes, including *optrA, poxtA, cfr*, or *cfr*(B) genes were detected in all *E. faecalis* UTI isolates, suggesting four *E. faecalis* UTI isolates, including one linezolid-intermediate isolate and three linezolid-resistant isolates, were found with positive carriage of the *optrA* gene and none was found with *poxtA, cfr*, or *cfr*(B) gene (shown in Table 3). Their features of four *optrA*-positive *E. faecalis* were shown in Table 3, indicating all three *E. faecalis* with linezolid resistance were detected with the carriage of *optrA* and just one linezolid-intermediate strain was positive. The genetic mutation of linezolid target sites, including the V domain of the 23S rRNA genes and 50S ribosome protein L3 and L4, were detected in linezolid-resistant/intermediate *E. faecalis*, indicating just G2576U genetic mutations in the V domain of the 23S rRNA genes were found in two linezolid-resistant *E. faecalis* isolates. That is to say, among the five linezolid-resistant *E. faecalis* found in this study, three *optrA-*positive isolates and the other two linezolid-resistant strains were explained by G2576U genetic mutations in the V domain of the 23S rRNA genes. The relationship of ST genotype with linezolid susceptibility, the carriage of several virulence factors and resistance genes in *E. faecalis* UTI isolates was shown in Table 4 and Supplementary Table 5. Overall, these *E. faecalis* isolates from UTI were classified into 21 ST types, in which the dominant clones were ST16 (35 strains, 30.43%) and ST179 (34 strains, 29.57%). Moreover, 31.42% (11/35) of ST16-*E. faecalis* and 2/34 (5.88%) of ST179-*E. faecalis* were not susceptible to linezolid.

**Table 4:** Relationship of MLST phenotype with linezolid susceptibility, virulence factors, and resistance genes.

**MLST**	**NO**	**Ratio (%)**	**Linezolid MIC distribution (μg/mL)**			***ace***	***efaA***	***asa1***	***cyl***	***esp***	***gelE***	***hyl***	***tet*(M)**	***erm*(A)**	***erm*(B)**	***erm*(C)**	***erm*(A) + *erm*(B)**	***erm*(A) + *erm*(C)**	***erm*(B) + *erm*(C)**
			**≤2**	**4**	**≥8**														
ST16	35	30.43	24	10	1	35	35	33	32	28	3	2	33	1	32	0	1	0	0
ST179	34	29.57	32	2	0	34	34	33	33	29	33	5	33	0	32	0	0	0	0
ST30	3	2.61	2	1	0	3	3	2	1	1	3	0	1	0	2	0	0	0	0
ST4	3	2.61	3	0	0	3	3	3	3	3	3	1	3	0	3	0	0	0	0
ST6	3	2.61	3	0	0	3	3	3	3	2	2	0	3	1	2	0	1	0	0
ST403	3	2.61	1	2	0	3	3	0	0	0	1	1	3	0	3	0	0	0	0
ST409	2	1.74	1	1	0	2	2	1	1	1	2	0	2	0	1	0	0	0	0
ST40	2	1.74	2	0	0	2	1	1	1	2	2	0	1	0	0	0	0	0	0
ST47	2	1.74	1	1	0	2	1	2	1	2	2	1	1	0	1	0	0	0	0
ST506	2	1.74	1	1	0	2	2	2	1	1	2	0	1	0	0	0	0	0	0
ST541	2	1.74	0	0	2	2	2	0	0	0	0	2	2	2	2	0	2	0	0

*ST, sequence type; +, means exist at the same strain*.

### Risk Factors for UTI With Linezolid-Resistant/Intermediate *E. faecalis* Isolates

The basic clinical data of the patients with *E. faecalis* UTI was shown in Table 5. Univariate analysis showed that indwelling catheter, trachea cannula catheter, *erm*(A) and *optrA* genes were the risk factors for patients with UTI caused by linezolid-resistant/intermediate *E. faecalis* isolates (*P* < 0.05; Table 5). Moreover, multivariable conditional logistic regression model indicated that indwelling catheter and trachea cannula should be considered as the independent predictors of linezolid-resistant/intermediate *E. faecalis* infections (Table 6).

**Table 5:** Univariate analysis of potential risk factors of linezolid-resistant/intermediate *E. faecalis*.

**Risk factor**	**Odds ratio**	**95% Confidence interval**	***P-*value**
Sex	1.400	0.614–3.193	0.423
Age	1.013	0.993–1.033	0.115
Tumor	1.797	0.473–6.824	0.384
Diabetes mellitus	0.606	0.213–1.726	0.345
Corticosteroid therapy	0.288	0.061–1.366	0.221
ICU admission	0.493	0.124–1.963	0.523
**Invasive procedures**			
Venous catheter	1.269	0.127–12.653	0.839
Indwelling catheter	0.415	0.181–0.951	**0.035**
D-J tube catheter	2.745	0.748–10.073	0.116
Trachea cannula catheter	0.092	0.023–0.363	** <0.001**
**Antibiotics therapy**			
Penicillin exposure	0.800	0.305–2.095	0.649
Cephalosporin exposure	0.835	0.363–1.920	0.671
Carbapenem exposure	0.301	0.076–1.201	0.162
Aminoglycoside exposure	0.413	0.025–6.792	0.523
Macrolideexposure	0.405	0.055–3.001	0.723
Tetracycline exposure	0.413	0.025–6.792	0.523
Glycopeptide exposure	2.075	0.186–23.169	0.545
Quinolone exposure	0.933	0.395–2.204	0.875
Antifungal agent exposure	1.030	0.972–1.092	0.121
Nitroimidazole exposure	1.030	0.972–1.092	0.121
Linezolid exposure	1.030	0.972–1.092	0.121
**Antibiotic resistance gene**			
*erm*(A)	0.038	0.004–0.332	** <0.001**
*erm*(B)	0.939	0.311–2.833	0.911
*tet*(M)	0.843	0.770–0.922	0.069
*optrA*	1.182	1.003–1.392	**0.002**

*Bold values mean statistically significant*.

**Table 6:** Multivariable conditional logistic regression model for potential risk factors of linezolid-resistant/intermediate *E. faecalis* UTI infection.

**Risk factor**	***P-*value**	***OR***	**95% *CI***
Indwelling catheter	0.03	5.297	1.178–23.816
Trachea cannula catheter	0.028	14.359	1.330–154.997

## Discussion

*E. faecalis* is one of the major causative pathogens of UTI among gram-positive bacteria (19). Due to acquired and intrinsic resistance, *E. faecalis* exhibits a high level of resistance to many commonly used antibiotics, including cephalosporin and macrolides. In this study, our data also indicated a high frequency of *E. faecalis* clinical isolates from UTI with antibiotics resistance toward tetracyclines, minocycline, and erythromycin. Worthy of attention, the frequency of *E. faecalis* UTI isolates with linezolid or tedizolid-resistant/intermediate *E. faecalis* isolates in this hospital setting was significantly high compared with other articles reported (20). Moreover, all *E. faecalis* UTI isolates in this study, including linezolid-resistant/intermediate isolates, remained still susceptible to ampicillin, vancomycin and tigecycline, indicating rarely cross resistance between linezolid and other antibiotics in *E. faecalis* clinical isolates isolated from this hospital setting. Our data also indicated that linezolid or tedizolid might not be the first-line choices of the antibiotics suitable for the antimicrobial treatment of *E. faecalis* infections in this hospital settings. Therefore, the resistance mechanism and risk factors of linezolid-resistant/intermediate *E. faecalis* infections deserve our attention and need to be further studied.

Several previous reports have indicated the clonality characteristics of linezolid resistance in Staphylococci and *E. faecalis* (21, 22). ST16 is the predominant STs of linezolid-resistant/intermediate *E. faecalis* clinical isolates in this study. Multiple reports have demonstrated that ST16 might become more adaptable to the hospital environment and acquire the multi-drug resistance (10, 13). Whereas, whether ST16 *E*. *faecalis* with linezolid resistance has been widely transmitted in this hospital setting or this district needs to be further studied.

The reports have indicated the outbreak of the high detection frequency of some linezolid-resistant gram-positive bacteria in the hospital settings and this may be explained by different causes, such as antibiotics exposure, environmental contamination factors, person-to-person contact transmission (23–25). The complex mechanism of linezolid resistance in *E*. *faecalis* can be explained by three mechanisms: (1) genetic mutations in linezolid target sites, including the domain V region of 23S rRNA genes; (2) mutations in *rpl*(D) or *rpl*(C) genes encoding 50S ribosomal proteins L3 and L4; and (3) acquisition and dissemination of the plasmid-borne genes *cfr, cfr*(B), *poxtA*, and ATP-binding cassette (ABC) transporter gene *optrA* (13, 16, 26, 27). The plasmid-borne genes *cfr* and *cfr*(B) haven't been found in this study and moreover, two linezolid-resistant strains have the G2576U genetic mutations in the V domain of the 23S rRNA, which is consistent with previous reports in our laboratory (28). Our data indicated the high carriage of *erm*(A) in linezolid-resistant/intermediate *E. faecalis* UTI isolates and three linezolid-resistant *E. faecalis* isolates were positive with *optrA*. The mechanism of *erm*(A) that participate in macrolide or clindamycin resistance is mainly mediated by methylating the V domain of 23S rRNA gene. No report supported linezolid resistance could be caused by *erm(*A) and we hypothesized linezolid-resistant/intermediate isolates might facilitate the carriage or transmission of this resistance gene in *E. faecalis*. Therefore, the correlation of *erm*(A) with linezolid susceptibility needs to be further studied. The plasmid-borne *optrA* can result in cross resistance to multiple antibiotics in gram-positive bacteria, including oxazolidinones (linezolid and tedizolid) and phenicols (13). The *optrA* gene was firstly demonstrated for the explanation of linezolid-resistant/intermediate *E. faecalis* and subsequently the rapid and transmission of this gene among *Enterococcus* spp. and other gram-positive bacteria was further reported worldwide (13, 15, 16, 23, 25). Recently, the carriage frequency of *optrA* in *Enterococcus* spp. from the animals of human food chain in China was reported to be higher than that from human host (15.9% vs. 2–2.9%, respectively) and then, this phenomenon was mainly explained by the continuous and wide application of florfenicol in the food-producing animals or the environment in China from 1999 (15, 29). Considering the high frequency of linezolid resistance in this study, we presume that the transmission of *optrA* may exist in the environment, food products, medical device surface and so on (30). Overall, the transmission routine and mechanism of *optrA* in linezolid-resistant/intermediate *E. faecalis* in this hospital setting should be further elucidated. It is worthy of note that one linezolid-intermediate strains was found *optrA* gene and it's still unknown for the mechanism explanation of linezolid-intermediate/resistance in majority of *E. faecalis* UTI isolates. We hypothesized that some unknown proteins or other resistance mechanisms, including the efflux pumps or some membrane proteins, might participate in the MIC enhancement of linezolid. Therefore, the mechanisms of linezolid-intermediate/resistance *E. faecalis* in this hospital setting need to be further studied. Some reports have shown that linezolid exposure is an independent risk factor for linezolid-resistant/intermediate *E. faecalis* infections in UTI (Case-control Studies) (31, 32). The univariate and multivariable conditional logistic regression of the risk factors of *E. faecalis* infection with linezolid resistance in this study suggested indwelling catheter and trachea cannula catheter as the independent predictors of linezolid-resistant/intermediate *E. faecalis* infections. It's well-known that indwelling catheter and trachea cannula are invasive operations in clinics and they are considered as one of the important causes of nosocomial infection, indicating the hospital environment and invasive operation might prompt the occurrence or dissemination of linezolid resistance in this hospital setting. However, linezolid exposure was not considered as a risk factor in this study, which could be explained by the narrow application of this drug in this hospital. Our data indicated that linezolid resistance, even in some medical environments without the wide application of linezolid, should be alert and might exhibit the high level due to the environmental transmission of linezolid-resistant/intermediate bacteria that possibly caused by invasive operations.

## Conclusion

Conclusively, this study demonstrated the high frequency of linezolid-resistant/intermediate *E. faecalis* in patients with UTI in this hospital setting. These isolates showed the characteristics of clonality to ST16 and ST179. Moreover, *E. faecalis* with linezolid resistance in this study might be explained by the high carriage frequency of *optrA* genes and the genetic mutation of linezolid target site. The invasive operations, especially indwelling catheter and trachea cannula catheter might facilitate the development of linezolid-resistant/intermediate *E. faecalis* UTI infection in hospital setting. The transmission routine of *optrA* in linezolid-resistant *E. faecalis* and the mechanisms of linezolid-intermediate/resistant *E. faecalis* in this hospital setting should be further elucidated.

## Data Availability Statement

The datasets presented in this study can be found in online repositories. The names of the repository/repositories and accession number(s) can be found in the article/Supplementary Material.

## Author Contributions

XM: participated in the design of the study, carried out RNA silencing test, analyzed, and interpreted the data, and drafted the manuscript. FZ and BB: performed antibiotic susceptibility testing, detected virulence genes by PCR, carried out the RNA silencing test, and participated in the data analysis. ZL and GX: conducted the MLST and CC analysis, and provided a critical revision of the manuscript. ZC, XS, and JZ: participated in the acquisition of the samples, isolated DNA, conducted MLST. QD and ZY: designed the study, participated in the data analysis, and provided critical revisions of the manuscript for important intellectual content. All authors contributed to the article and approved the submitted version.

## Conflict of Interest

The authors declare that the research was conducted in the absence of any commercial or financial relationships that could be construed as a potential conflict of interest.
