# Supplementary material for: Linezolid Resistance in Enterococcus faecalis Associated With Urinary Tract Infections of Patients in a Tertiary Hospitals in China: Resistance Mechanisms, Virulence, and Risk Factors
Source: Front Public Health. 2021 Feb 5;9:570650. doi: 10.3389/fpubh.2021.570650 (PMC7893085; doi:10.3389/fpubh.2021.570650)
Supplement: Supplementary file 1 [file Data_Sheet_1.docx]

**Supplement**





FigS1 ward orignin of clinical *E.faecalis* isolates. Note: 60 strains (52.17%) came from Urinary surgery Department, followed by 10 strains (8.70%) in Oncology department, 8 strains (6.96%) in Nephrology department, 6 strains (5.22%) in ICU,4 strains (3.48%) in Rehabilitation department, 3 strains (2.61%) in Neurology department, Cardiovascular department, Infection department and Emergency department, and 12 strains (10.43%) in other departments.





**Figure S2. Distribution characteristics of virulence genes in *E. faecalis***

**Table S1 Primers used to detect targeted resistance genes by PCR**

| **Target gene** | **Primer** | **Primer sequence (5'-3')** | **Amplicon size(bp)** | **References** |
| --- | --- | --- | --- | --- |
| 23S rRNA | 23S-F | GGC GCT GGT GGG ATA CTA | 1898 | Bai B et al 2018 |
|  | 23S-1R | GGA CGG TTA TGA GCC GTC |  |  |
|  | 23S-F | GGC GCT GGT GGG ATA CTA | 1415 |  |
|  | 23S-2R | GCG ATC TCC TGC GTG AC |  |  |
|  | 23S-F | GGC GCT GGT GGG ATA CTA | 1111 |  |
|  | 23S-3R | CCC TTC TTC AAG CTT ATC |  |  |
|  | 23S-F | GGC GCT GGT GGG ATA CTA | 1952 |  |
|  | 23S-4R | CCA CAG TGA TTT TGC CCA |  |  |
|  | 23S-V/F | AGT TTG ACT GGG GCG GTC | 429 |  |
|  | 23S-V/R | CCG GTC CTC TCG TAC TA |  |  |
| Ribosomal | L3/L4-F | TAC CAA CAG AAC GCT CAC | 4323 | Locke JB et al., 2009 |
| L3/L4 | L3/L4-R | CCT TTA CCT TTA CCC ATA C |  |  |
| *erm*(A) | *ermA*-F | TCTAAAAAGCATGTAAAAGAAA | 553 | Bai B et al 2018 |
|  | *ermA*-R | CGATACTTTTTGTAGTCCTTC |  |  |
| *erm*(B) | *ermB*-F | CCGTTTACGAAATTGGAACAGGTAAAGGGC | 359 | Bai B et al 2018 |
|  | *ermB*-R | GAATCGAGACTTGAGTGTGC |  |  |
| *erm*(C) | *ermC*-F | GCTAATATTGTTTAAATCGTCAATTCC | 460 | Bai B et al 2018 |
|  | *ermC*-R | GGATCAGGAAAAGGACATTTTAC |  |  |
| *tet*(M) | *tet M*-F | CAATACAATAGGAGCAAGC | 974 | Bai B et al 2018 |
|  | *tet M*-R | CGAACAAGAGGAAAGCATAAG |  |  |
| *optrA* | *optrA*-F | AGG TGG TCA GCG AAC TAA | 1395 | Wang Y et al., 2015 |
|  | *optrA*-R | ATC AAC TGT TCC CAT TCA |  |  |
| *cfr* | *cfrA*-F | TGA AGT ATA AAG CAG GTT GGG AGT | 746 | Bai B et al 2018 |
|  | *cfrA*-R | ACC ATA TAA TTA CCA CAA GCA GC |  |  |
| *cfr*(B) | *cfrB-*F | ATA ACG GTT CTT CCT AAA TCA CTA A | 1395 | Deshpande LM, et al. 2015 |
|  | *cfrB-*R | CAT CTA ATG TAT CCA TCA CAT CTG |  |  |
| *poxtA* | *poxtA*-F | TATTGTCGGCGTGAACGGAG | 1355 | Egan S A, et al.2020 |
|  | *poxtA*-R | TCTGCGTTTCTGGGTCAAGG |  |  |

**Table S2 Primers used to detect targeted virulence genes by PCR**

| **Target** | **Primer** | **Primer sequence (5'-3')** | **Amplicon size (bp)** | **References** |
| --- | --- | --- | --- | --- |
| *esp* | *esp*-F | AATTGATTCTTTAGCATCTGG | 510 | Vankerckhoven V, et al. 2004 |
|  | *esp*-R | AGATTCATCTTTGATTCTTGG |  |  |
| *asal* | *asal*-F | GCACGCTATTACGAACTATGA | 375 | Vankerckhoven V, et al. 2004 |
|  | *asal*-R | TAAGAAAGAACATCACCACGA |  |  |
| *hyl* | *hyl*-F | GACTGACGTCCAAGTTTCCAA | 276 | Vankerckhoven V, et al. 2004 |
|  | *hyl*-R | ACAGAAGAGCTGCAGGAAATG |  |  |
| *cyl* | *cyl*-F | ACTCGGGGATTGATAGGC | 688 | Vankerckhoven V, et al. 2004 |
|  | *cy*l-R | GCTGCTAAAGCTGCGCTT |  |  |
| *gelE* | *gelE*-F | TATGACAATGCTTTTTGGGAT | 213 | Vankerckhoven V, et al. 2004 |
|  | *gelE*-R | AGATGCACCCGAAATAATATA |  |  |
| *efaA* | *efaA*-F | GCCAATTGGGACAGACCCTC | 688 | Barbosa-Ribeiro M, et al. 2016 |
|  | *efaA*-R | CGCCTTCTGTTCCTTCTTTGGC |  |  |
| *ace* | *ace*-F | AAAGTAGAATTAGATCCACAC | 320 | Bai B et al. 2018 |
|  | *ace*-R | TCTATCACATTCGGTTGCG |  |  |

**Table S3 The Primers used to amplify house-keeping genes by PCR in *E.faecalis* MLST**

| **Target** | **Primer** | **Primer sequence (5'-3')** | **Amplicon size (bp)** | **References** |
| --- | --- | --- | --- | --- |
| *gdh* | *gdh*-F | GGCGCACTAAAAGATATGGT | 530 | Bai B et al. 2018 |
|  | *gdh*-R | CCAAGATTGGGCAACTTCGTCCCA |  |  |
| *gyd* | *gyd*-F | CAAACTGCTTAG CTCCAATGGC | 395 | Bai B et al. 2018 |
|  | *gyd*-R | CATTTCGTTGTCATACCAAGC |  |  |
| *pstS* | *pstS*-F | CGGAACAGGACTTTCGC | 583 | Bai B et al. 2018 |
|  | *pstS*-R | ATTTACATCACGTTCTACTTGC |  |  |
| *gki* | *gki*-F | GATTTTGTGGGAATTGGTATGG | 438 | Bai B et al. 2018 |
|  | *gki*-R | ACCATTAAAGCAAAATGATCGC |  |  |
| *aroE* | *aroE*-F | TGGAAAACTTTACGGAGACAGC | 459 | Bai B et al. 2018 |
|  | *aroE*-R | GTCCTG TCCATTGTTCAAAAGC |  |  |
| *xpt* | *xpt*-F | AAAATGATGGCCGTGTATTAGG, | 456 | Bai B et al. 2018 |
|  | *xpt*-R | AACGTCACCGTTCCTTCACTTA |  |  |
| *yqiL* | *yqiL*-F | CAGCTTAAGTCAAG TAAGTGCCG | 436 | Bai B et al. 2018 |
|  | *yqiL*-R | GAATATCCCTTCTGCTTGTGCT |  |  |

**Table S4 Overall resistance and sensitivity of *E. faecalis* isolates against tested antibiotics**

| Antibiotic | Sensitivity | |  | Intermediate | |  | Resistance | |
| --- | --- | --- | --- | --- | --- | --- | --- | --- |
|  | No.of strains | Rate(%) |  | No.of strains | Rate(%) |  | No.of strains | Rate(%) |
| Tetracycline | 8 | 6.96% |  | 3 | 2.61% |  | 104 | 90.43% |
| Doxycycline | 7 | 6.09% |  | 7 | 6.09% |  | 101 | 87.83% |
| Minocycline | 9 | 7.83% |  | 12 | 10.43% |  | 94 | 81.74% |
| Erythromycin | 1 | 0.87% |  | 19 | 16.52% |  | 95 | 82.61% |
| Tigecycline | 115 | 100% |  | - | - |  | 0 |  |
| Vancomycin | 115 | 100% |  | 0 |  |  | 0 |  |
| Tedizolid | 100 | 86.96% |  | - | - |  | 15 | 13.04% |
| Linezolid | 89 | 77.39% |  | 21 | 18.26% |  | 5 | 4.35% |

**Table S5 Relationship of MLST phenotype with LZD susceptibility, virulence genes and resistance genes**

| MLST | NO | Ratio  (%) | Linezolid MIC distribution  (μg/mL) | | | *ace* | *efaA* | *asa1* | *cyl* | *esp* | *gelE* | *hyl* | *tet*(M) | *erm*(A) | *erm*(B) | *erm*(C) | *erm*(A)  +  *erm*(B) | *erm*(A)  +  *erm*(C) | *erm*(B)+  *erm*(C) |
| --- | --- | --- | --- | --- | --- | --- | --- | --- | --- | --- | --- | --- | --- | --- | --- | --- | --- | --- | --- |
|  |  |  | ≤2 | 4 | ≥8 |  |  |  |  |  |  |  |  |  |  |  |  |  |  |
| **ST139** | 1 | 0.87 | 1 | 0 | 0 | 1 | 1 | 1 | 1 | 1 | 1 | 0 | 1 | 0 | 1 | 0 | 0 | 0 | 0 |
| **ST207** | 1 | 0.87 | 1 | 0 | 0 | 1 | 1 | 1 | 1 | 0 | 1 | 1 | 1 | 0 | 0 | 0 | 0 | 0 | 0 |
| **ST7480** | 1 | 0.87 | 1 | 0 | 0 | 1 | 1 | 1 | 0 | 1 | 0 | 0 | 1 | 0 | 1 | 0 | 0 | 0 | 0 |
| **ST585** | 1 | 0.87 | 1 | 0 | 0 | 1 | 1 | 1 | 1 | 1 | 1 | 0 | 1 | 0 | 1 | 0 | 0 | 0 | 0 |
| **ST414** | 1 | 0.87 | 1 | 0 | 0 | 1 | 1 | 0 | 0 | 0 | 1 | 0 | 0 | 0 | 1 | 0 | 0 | 0 | 0 |
| **ST300** | 1 | 0.87 | 1 | 0 | 0 | 1 | 1 | 1 | 0 | 0 | 0 | 0 | 0 | 0 | 1 | 0 | 0 | 0 | 0 |
| **ST63** | 1 | 0.87 | 0 | 1 | 0 | 1 | 1 | 0 | 0 | 0 | 1 | 0 | 1 | 0 | 1 | 0 | 0 | 0 | 0 |
| **ST34** | 1 | 0.87 | 1 | 0 | 0 | 1 | 1 | 0 | 0 | 0 | 1 | 1 | 1 | 0 | 0 | 0 | 0 | 0 | 0 |
| **ST480** | 1 | 0.87 | 1 | 0 | 0 | 1 | 1 | 1 | 0 | 0 | 0 | 1 | 1 | 0 | 0 | 0 | 0 | 0 | 0 |
| **NT** | 15 | 13.04 | 11 | 2 | 2 | 15 | 15 | 10 | 8 | 7 | 14 | 9 | 11 | 3 | 8 | 0 | 2 | 0 | 0 |

**ST：sequence type, NT:non-typeable,+:means exist at the same strain**
